# Atorvastatin and Vitamin E Accelerates NASH Resolution by Dietary Intervention in a Preclinical Guinea Pig Model

**DOI:** 10.3390/nu11112834

**Published:** 2019-11-19

**Authors:** Julie Hviid Klaebel, Mia Skjødt, Josephine Skat-Rørdam, Günaj Rakipovski, David H. Ipsen, Anne Marie V. Schou-Pedersen, Jens Lykkesfeldt, Pernille Tveden-Nyborg

**Affiliations:** 1 Department of Veterinary and Animal Sciences, Faculty of Health and Medical Sciences, University of Copenhagen, Ridebanevej 9, 1870 Frederiksberg C, Denmark; juliehviid@sund.ku.dk (J.H.K.); huaiaus@hotmail.com (M.S.); jsr@sund.ku.dk (J.S.-R.); dhi@sund.ku.dk (D.H.I.); am.schoupedersen@sund.ku.dk (A.M.V.S.-P.); jopl@sund.ku.dk (J.L.); 2 CV Research, Global Research, Novo Nordisk A/S, Novo Nordisk Park 1, 2760 Måløv, Denmark; GURA@novonordisk.com

**Keywords:** Non-alcoholic fatty liver disease, Non-alcoholic steatohepatitis, vitamin E, atorvastatin, lifestyle modifications, hepatic lesions, fibrosis

## Abstract

Despite affecting millions of patients worldwide, no pharmacological treatment has yet proved effective against non-alcoholic steatohepatitis (NASH) induced liver fibrosis. Current guidelines recommend lifestyle modifications including reductions in dietary energy intake. Recently, therapy with atorvastatin and vitamin E (vitE) has been recommended, although clinical studies on the resolution of hepatic fibrosis are inconclusive. Targeting NASH-induced hepatic end-points, this study evaluated the effects of atorvastatin and vitE alone or in combination with a dietary intervention in the guinea pig NASH model. Guinea pigs (*n* = 72) received 20 weeks of high fat feeding before allocating to four groups: continued HF feeding (HF), HF diet with atorvastatin and vitE (HF+), low-fat diet (LF) and low-fat with atorvastatin and vitE (LF+), for four or eight weeks of intervention. Both LF and LF+ decreased liver weight, cholesterol and plasma dyslipidemia. LF+ further improved hepatic histopathological hallmarks (*p* < 0.05), liver injury markers aspartate aminotransferase (AST) and alanine aminotransferase (ALT) (*p* < 0.05) and reduced the expression of target genes of hepatic inflammation and fibrosis (*p* < 0.05), underlining an increased effect on NASH resolution in this group. Collectively, the data support an overall beneficial effect of diet change, and indicate that atorvastatin and vitE therapy combined with a diet change act synergistically in improving NASH-induced endpoints.

## 1. Introduction

Non-alcoholic fatty liver disease (NAFLD) has emerged as a leading cause of chronic liver disease, estimated to have a global prevalence of 25% in adults and is currently the second leading indication of liver transplantations in the United States [1]. NAFLD is considered the hepatic manifestation of the metabolic syndrome covering a wide spectrum of conditions ranging from non-alcoholic fatty liver to non-alcoholic steatohepatitis (NASH) [2]. Fueled by a continuous intake of a westernized diet, high in fat and sugars, a vicious cycle of dyslipidemia, hepatic lipid overload and metabolic imbalance induces a chronic state of hepatic steatosis. This increases the risk of progression to steatohepatitis, hallmarked by steatosis with accompanying hepatic inflammation and hepatocellular injury (ballooning hepatocytes) damaging the hepatic tissue [3]. The formation of intrahepatic fibrosis is a severe risk factor for cirrhosis, hepatocellular carcinoma and life threatening liver failure [4]. In overall and liver-related mortality in NAFLD patients, fibrosis stage has proved to be a superior predictor. Therefore, targeting fibrosis resolution plays a central role in the investigation of disease management [5,6,7].

No pharmacological therapies targeting pathophysiological disease modalities are approved for NAFLD or NASH and with the predicted increase in prevalence, the need for an effective pharmacological intervention strategy is urgent [1]. Currently, treatment of NASH is limited to lifestyle modifications and management of comorbidities such as dyslipidemia and weight reduction [8]. Standard care of NASH in the United States often consists of a combination therapy with atorvastatin and vitamin E (vitE) [9,10,11]. Although there are no clinical data supporting the efficacy on regression of fibrosis, positive results in the management of dyslipidemia and resolution of NASH have been shown [9,10]. 

Atorvastatin acts as a lipid-lowering agent by inhibiting the 3-hydroxy-3-methylglutaryl-coenzyme A reductase (HMGR), the rate-limiting enzyme in cholesterol synthesis, and has been shown to reduce plasma aminotransferases, low-density lipoprotein, and total plasma cholesterol, and improve hepatic steatosis and inflammation [12,13]. As an antioxidant, vitE has been suggested to improve redox homeostasis by decreasing the level of reactive oxygen species and preventing oxidative damage [14]. Meta-analyses of double blinded, single blinded and open label clinical trials show vitE to decrease aminotransferase levels and to improve hepatic histopathology by reducing steatosis, lobular inflammation and hepatocellular ballooning [15,16]. VitE may exhibit anti-fibrotic properties by suppressing the expression of transforming growth factor β (TGF-β), thereby inhibiting the activation of hepatic stellate cells (HSC) central for the formation of fibrous extracellular matrix; however, clinical studies have not been conclusive [15,17].

Applying the validated guinea pig NASH model with hepatic fibrosis [3,18], this study investigated the effect of atorvastatin and vitE on dyslipidemia, histopathological hallmarks of NASH and molecular pathogenic drivers of the disease [19]. Effects of atorvastatin and vitE intervention in doses corresponding to those in human patients were explored in combination with or without a diet change, mimicking the general lifestyle modifications that accompany clinical treatment advice in NAFLD/NASH. Our hypothesis was that combining lifestyle changes with atorvastatin and vitE would result in a synergistic effect on the resolution of NASH.

## 2. Materials and Methods 

### 2.1. Animals and Experimental Design

All animal experimentation was approved by the Animal Experimentation Inspectorate under the Danish Ministry of Environment and Food, and in accordance with the European Legislation of Animal Experimentation 2010/63/EU. Group sizes were calculated by power analysis with variances adopted from our previous studies (Ipsen et al.) [18] and an effect size of 30% considered as therapeutically relevant.

Seventy-two female Hartley guinea pigs (weighing 450–500 g; Envigo, NM Horst, The Netherlands) were tagged subcutaneously with a microchip (E-vet, Haderslev, Denmark) for identification upon arrival. Animals were group-housed in floor pens with wood shavings, hay, straw and environmental enrichment, ad libitum access to feed and water, and kept on a 12-h light–dark cycle with temperatures of 20–24 °C. Throughout the study, animal caretakers inspected animals daily and body weights were recorded weekly. 

Following one week of acclimatization on a standard guinea pig chow diet, baseline blood samples were collected from 12 randomly selected animals, before placing all animals on a high fat (HF) diet (20% fat, 0.35% cholesterol, and 15% sucrose) for 20 weeks of preload period. To provide preload baseline values, eight randomly selected animals were euthanized at Week 20, immediately before commencing the intervention. The remaining guinea pigs were weight stratified into four groups (n = 16): a high-fat group (HF) continuing on the HF diet, a high-fat group treated with atorvastatin and vitE (HF+; 20 mg/kg feed atorvastatin and 375 mg/kg feed all-rac vitE, in guinea pigs corresponding to a daily intake of 1 mg/kg atorvastatin and 800 IU vitE and consistent with doses of atorvastatin and vitE administered to human patients), a low fat group (LF) receiving a chow diet (4% fat, 0% cholesterol, and 0% sucrose) or a LF+ group receiving a chow diet with atorvastatin and vitE (LF+, 20 mg/kg feed atorvastatin and 375 mg/kg feed vitE). All diets were manufactured by Ssniff (Ssniff Spezialdiäten, Soest, Germany, Table 1) and stored at minus 20 °C upon arrival. Feed was thawed twice weekly in small batches to reduce auto-oxidation. Atorvastatin was purchased from Sigma-Aldrich (Sigma-Aldrich Chemie GmbH, Schnelldorf, Germany) and vitE supplied by Ssniff. Overall Diet composition can be seen in Table 1.

**Figure 1:**
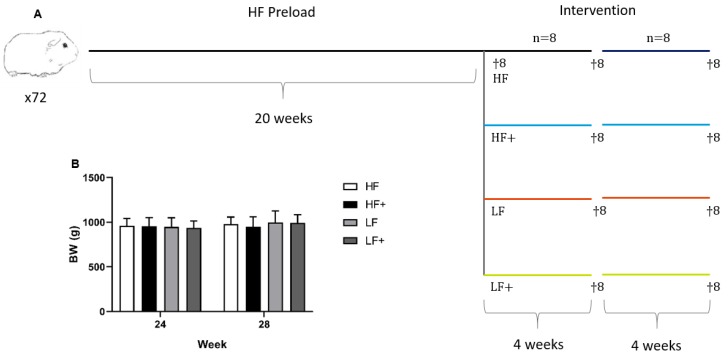
Study overview and body weights of animals. (**A**) Seventy-two guinea pigs were preloaded on a high-fat diet for 20 weeks. Prior to intervention, eight animals were randomly selected for euthanasia, serving as baseline values. The remaining guinea pigs were weight stratified into the following groups (*n* = 16): HF, continuing on the high-fat diet (20%, 0.35% cholesterol, and 15% sucrose); HF+, receiving high-fat diet with atorvastatin and vitamin E (20 mg/kg feed atorvastatin and 375 mg/kg feed all-rac vitamin E, for a guinea pig corresponding to a daily intake of 1 mg/kg atorvastatin and 800 IU vitamin E); LF, receiving a standard guinea pig chow diet (4% fat, 0% cholesterol, and 0% sucrose); LF+, receiving a standard guinea pig chow diet with atorvastatin and vitamin E (20 mg/kg feed atorvastatin and 375 mg/kg feed vitamin E). After four weeks of intervention, eight randomly chosen guinea pigs, from each group, were euthanized, leaving the remaining eight animals for another four weeks of intervention before termination. (**B**) No differences were recorded in body weight (BW) through the study.

After four weeks of intervention (Week 24), eight randomly chosen guinea pigs from each group were euthanized, leaving the remaining animals (n = 8/group) to continue for another four weeks before termination (Week 28). A study overview and body weights of the animals are shown in Figure 1. Euthanasia was done as previously described (Tveden-Nyborg et al.; Ipsen et al.) [3,18]. In short, guinea pigs were semi-fasted overnight (access to hay but not feed), pre-anaesthetized with 1.25 mL/kg body weight Zoletil-mix (zoletil-50 supplemented with xylazine and butophanol) and placed on 5% isoflurane [3]. Once interdigital reflexes had disappeared, the thoracic cavity was opened and an intracardial blood sample was collected and immediately followed by euthanasia by decapitation.

### 2.2. Plasma Samples

Before initiating the HF feeding preload period, blood samples were obtained from 12 randomly chosen animals (“Week 0”). Samples were collected from the vena saphena in K_3_-EDTA microvettes (Sarstedt, Numbrecht, Germany), centrifuged and plasma isolated and stored at minus 20 °C. Samples were afterwards analyzed for total cholesterol (TC), triglyceride (TG), aspartate aminotransferase (AST) and alanine aminotransferase (ALT) on a Cobas 6000 (see below).

At euthanasia, intracardial blood samples were collected on anaesthetized animals as previously described (Tveden-Nyborg et al.) [3] in K_3_-EDTA flushed 10 mL syringes, with the exception of samples collected for analyses of free fatty acids (FFA) and alkaline phosphatase (ALP), which were collected in sodium fluoride (NaF) and heparin coated microvettes (Sarstedt, Nümbrecht, Germany), respectively. 

For all blood samples, plasma was isolated immediately following collection, by centrifuging at 2000 g for 4 min at 4 °C. Analyses of TC, TG, AST, ALT, FFA and ALP were performed on a Cobas 6000 (Roche Diagnostic Systems, Berne, Switzerland) according to manufacturer’s instructions. Markers of oxidative stress: total ascorbic acid (TAA; plasma stabilized in metaphosphoric acid), alpha-tocopherol/vitE (αToc), dihydrobiopterin (BH_2_; blood stabilized with dithioerythritol) and tetrahydrobiopterin (BH_4_; blood stabilized with dithioerythritol) were analyzed by HPLC as previously described (Lykkesfeldt; Reimann et al.; Mortensen et al.; Hissin and Hilf) [20,21,22,23,24]

### 2.3. Liver Samples

Upon excision, the liver was rinsed in ice cold PBS, gently dried on paper cloth before weighing and being photographed. Seven 0.3–0.5 cm thick samples were isolated with a scalpel from the left lateral lobe (lobus hepatis sinister lateralis) with one section designated to biochemical, four sections to molecular biological and two sections to histological analyses, respectively. 

Liver sections for biochemical analyses were frozen on dry ice and stored at minus 80 °C. Afterwards, samples were homogenized and TC and TG content analyzed on a Cobas 6000 as described previously (Tveden-Nyborg et al.) [3]. Briefly, 30–40 mg tissue were homogenized with 1.0 mL extraction buffer (0.15 M sodium acetate and 0.75% Triton-X). The homogenates were placed in a 100 °C heating block for 2 min before being cooled on ice and supplemented with 0.5 mL extraction buffer. Samples were centrifuged at 9000 g for 10 min at 4 °C and the supernatant collected for analyses of TC and TG levels.

Levels of liver oxidative stress markers, namely αToc, AA, BH_2_, BH_4_, L-arginine (L-arg) and asymmetric dimethylarginine (ADMA), were measured by HPLC, as mentioned above. Glutathione (GSH) and oxidized glutathione (GSSG) were measured according to (Hissin and Hilf) [24] and malondialdehyde (MDA) measured according to (Lykkesfeldt) [25]. Using colorimetry (Randox Superoxide Dismutase (Ransod assay), Randox Laboratories Ltd, Crumlin, UK), superoxide dismutase (SOD) was measured according to manufacturer’s specifications. Liver samples for αToc analysis were stabilized with butylated hydroxytoluene and measured according to Burton et al. [26].

### 2.4. Histology

Liver sections were fixated in 10% formalin, before processing and embedding in paraffin and cut into 2–4 µm thick sections. The liver sections were stained with hematoxylin and eosin (H&E) and Masson’s trichrome (MT) and scored in a randomized and blinded fashion according to the guidelines of the semi-quantitative scoring system developed by Kleiner et al. [18,27]. Steatosis, inflammation and ballooning hepatocytes was scored on H&E stained sections, while fibrosis was scored on MT stained sections. The degree of steatosis was assessed across the entire liver section and graded as 0 (< 5%), 1 (5–33%), 2 (> 33–66%) or 3 (> 66%), while lobular inflammation and ballooning hepatocytes was examined in 20 randomly selected fields (20× zoom) of view in each liver section using the Visiopharm software package (Visiopharm, version 2018.9.4.5608, Visiopharm A/S, Hørsholm, Denmark). Lobular inflammation was graded as average number of foci pr. field as 0 (not present), 1 (< 2 foci), 2 (2–4 foci) or 3 (> 4 foci), with a focus being ≥ 3 inflammatory cells, and ballooning hepatocytes as 0 (not present), 1 (few ballooning hepatocytes) or 2 (many/prominent ballooning hepatocytes). Fibrosis was evaluated across the entire liver section and scored as 0 (not present), 1 (mild, zone 3, perisinusoidal; moderate, zone 3, perisinusiodal; portal/periportal), 2 (perisinusiodal and portal/periportal), 3 (bridging) and 4 (cirrhosis). Portal inflammation was assessed in three intact portal areas distributed on the liver section (i.e., left, center and right side of the section) as 0 (not present) or 1 (present), where presence was defined by ≥ 2 portal areas containing ≥ 2 inflammatory foci, with a focus defined as a cluster of ≥ 5 inflammatory cells. The NAFLD activity score (NAS) [27] was calculated as the sum of steatosis, inflammation and ballooning hepatocytes ranging from 0 to 8. At Week 28, liver morphology was scored as 0 (normal), 1 (mild; conserved liver architecture with altered hepatocyte morphology in Zone 3), 2 (moderate; compromised liver architecture *or* altered hepatocyte morphology in Zones 3 *and* 1 and/or Zone 2), 3 (severe; compromised liver architecture *and* altered hepatocyte morphology in Zones 3 *and* 1 and/or Zone 2) or 4 (very severe; complete loss of normal liver architecture *and* altered hepatocyte morphology in Zones 3 *and* 1 and/or Zone 2).

### 2.5. qPCR

Samples isolated for qPCR analysis were snap frozen in liquid nitrogen and stored at minus 80 °C until use. RNA extraction, reverse transcription and execution of qPCR were done according to Ipsen et al. [19]. Briefly, 50 mg of liver tissue were homogenized in 1000 µL MagMax Lysis/Binding Solution Concentrate (Thermo Fisher, Waltham, MA, USA) with 0.7% β-mercaptoethanol (Sigma Aldrich, MO, USA) and centrifuged for 1 min at 10,000 RPM and 4 °C. The supernatant was isolated and transferred to RNAse-free Eppendorf tubes and stored at minus 20 °C for 24 h. RNA was isolated using the MagMax-96 Total RNA Isolation Kit (Thermo Fisher, Waltham, MA, USA). Subsequently, cDNA was synthesized by subjecting 500 ng RNA to reverse transcription (High Capacity cDNA Reverse Transcription Kit; Thermo Fisher, Waltham, MA, USA) on a 2720 Thermal Cycler (Applied Biosystems, Foster City, CA, USA) under the following conditions: 25 °C for 10 min, 37 °C for 120 min, and 85 °C for 5 s. An intron-spanning primer set (β-actin) was applied to check the cDNA for genomic DNA contamination prior to further analyses, as done previously (Tveden-Nyborg et al.) [28]. In the qPCR analyses, 2 µL of undiluted cDNA were mixed with 8 µL Master Mix (5 µL PowerUp SYBR Green Master Mix (Thermo Fisher, Waltham, MA, USA), 1 µL Primer mix and 2 µL RNAse-free water). All samples were run in triplicates on the StepOnePlus^TM^ Real Time PCR system (Applied Biosystems, Foster City, CA, USA) under the following conditions: 50 °C for 2 min, 95 °C for 5 min and then 40 cycles consisting of 95 °C for 10 s, 60 °C for 10 s and 72 °C for 20 s. For tumor necrosis factor α (TNFα) and interleukin 8 (IL-8), the annealing step was executed at 62 °C instead of 60 °C. Melting curves were inspected for each primer set and hypoxanthine phosphoribosyltransferase 1 was used as reference gene. Primer information is listed in Table 2.

### 2.6. Statistical Analyses

Statistical analyses were performed in GraphPad Prism 8 (GraphPad Software, La Jolla, CA, USA) and SAS Enterprise Guide 7.1 (SAS Institute Inc, Cary, NC, USA). Levels of plasma TC, TG, ALT and AST at Week 20 were analyzed against Week 0 with an unpaired students T-test.

Markers of plasma and hepatic lipids, liver injury, oxidative stress and liver weight (LW) were analyzed by two-way ANOVA and analyses on expression of target genes were performed on ∆∆CT-values with one-way ANOVA, with multiple comparisons corrected by Tukey’s post hoc test. Data are presented as means with standard deviations (SD). Liver histology data were analyzed by the non-parametric Kruskal–Wallis test with Dunn’s post hoc test for multiple comparisons and are presented as individual values with medians. If heterogeneous variances were observed, datasets were log-transformed before analysis and then back-transformed and presented as geometric means with 95% confidence intervals. In the case of multiple *p*-values, only one is reported with one of the following overall values: *p* < 0.05, *p* < 0.01, *p* < 0.001 and *p* < 0.0001. If only one *p*-value, the exact value is reported. A *p*-value below 0.05 was considered statistically significant.

## 3. Results

### 3.1. Plasma and Hepatic Vitamin E Status

Analyzing plasma vitE values by two-way ANOVA showed that both diet and therapy significantly caused an increase in vitE plasma levels at Weeks 24 and 28 (*p* < 0.05; Table 3). Furthermore, levels of plasma vitE were significantly increased in HF+ compared with LF+ for both time points (*p* < 0.05; Table 3). After 24 weeks, no effect of diet or treatment was detected by two-way ANOVA analysis of hepatic vitE levels. However, continuing for 28 weeks showed a significant effect of treatment (*p* < 0.001), with both HF+ and LF+ vitE levels significantly elevated compared with HF and LF (*p* < 0.05; Table 4 and Table 5).

### 3.2. Dyslipidemia

**Figure 2:**
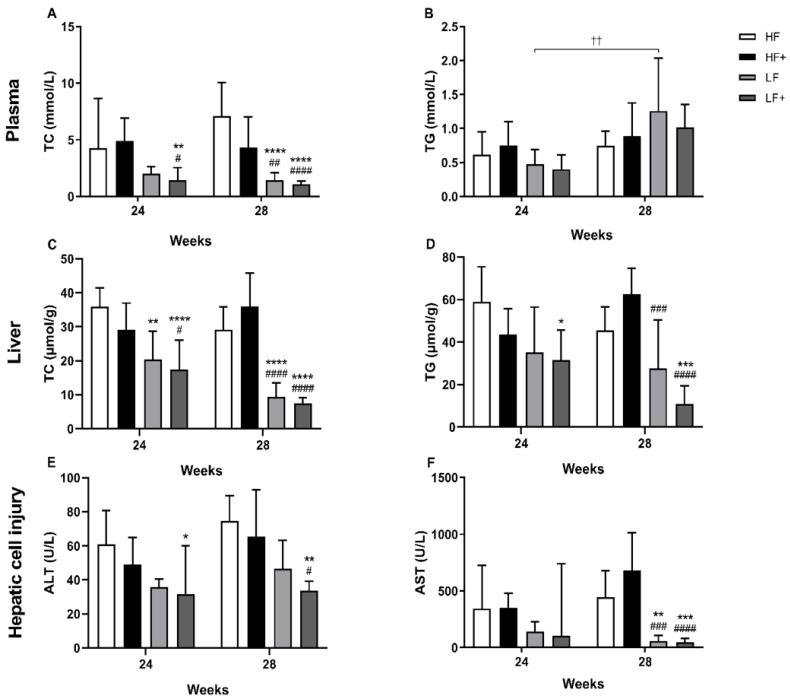
Evaluation of plasma and hepatic TC and TG levels and status of hepatic cell injury. (**A**) LF+ were significantly decreased compared with HF and HF+ after 24 weeks. After 28 weeks, both LF and LF+ were significantly reduced compared with HF and HF+. (**B**) The level of TG in the LF group significantly increased over time. No further differences were detected between groups, although data indicate an increase in TG level in both LF and LF+ over time. (**C**) Within 24 weeks, LF and LF+ showed significantly reduced TC levels compared with HF. Furthermore, LF+ were also significantly reduced compared with HF+. After 28 weeks, LF and LF+ were significantly reduced compared with HF and HF+. (**D**) The LF+ group showed decreased TG levels compared with HF after 24 weeks and decreased TG levels compared with HF and HF+ after 28 weeks. The LF group were significantly reduced compared to HF+ after 28 weeks. (**E**) Reduced levels of ALT were detected in the LF+ group compared with HF after 24 weeks and compared with HF and HF+ after 28 weeks. (**F**) Both LF and LF+ displayed reduced AST levels compared with HF and HF+ after 28 weeks. Plasma TC and TG; Liver TC and TG, data are presented as mean ± standard deviation. ALT; AST, data are presented as geometric mean with 95% confidence intervals. * *p* < 0.05, ** *p* < 0.01, *** *p* < 0.001, **** *p* < 0.0001, compared with HF # *p* < 0.05, ## *p* < 0.01, ### *p* < 0.001, #### *p* < 0.0001, compared with HF+ †† *p* < 0.01, LF Week 28 compared with LF Week 24. TC, total cholesterol; TG, triglyceride; ALT, alanine aminotransferase; AST, aspartate aminotransferase.

Levels of plasma TC (Week 0: 1.24 ± 0.29 vs. Week 20: 8.05 ± 2.90) confirmed dyslipidemia after 20 weeks of HF feeding (pre-intervention), compared with Week 0 (*p* < 0.001). An increase in TG levels (Week 0: 0.68 ± 0.25 vs. Week 20: 0.96 ± 0.35) was also observed, although not reaching significance (*p* = 0.05). After 24 and 28 weeks, LF+ had significantly lower levels of TC compared with HF and HF+ at both Weeks 24 and 28 (*p* < 0.01). LF were only significantly different from HF and HF+ after 28 weeks (*p* < 0.01). Levels of TG in LF and LF+ animals appeared to be increasing over time, however only the LF group was significantly different from Week 24 to 28 (*p* = 0.0073; Figure 2). No significant differences were observed in FFA levels between groups or over time (Table 3). 

### 3.3. Biochemical Markers

Plasma samples: A significant increase in the liver injury enzymes, ALT (Week 0: 31.83 ± 5.6 vs. Week 20: 49.58 ± 9.97) and AST (Week 0: 34.52 (28.86–40.17) vs. Week 20: 251.65 (160.40–342.90)), from Week 0 to Week 20 of HF feeding, was observed (*p* < 0.0001) indicating hepatic cell injury. Introducing a diet change and pharmacological intervention resulted in significant decrease of ALT levels in the LF+ group compared with the HF group after 24 weeks (*p* = 0.0220), while no effect was seen on the AST levels. After 28 weeks, levels of ALT were significantly reduced in LF+ compared with HF and HF+ (*p* < 0.05). Furthermore, both LF and LF+ showed significantly reduced AST levels (*p* < 0.01) at this time point compared with HF and HF+ (Figure 2). ALP levels were shown to be elevated in LF and LF+ compared with HF and HF+, with a significant effect of diet (*p* < 0.05; Table 3)

To assess oxidative stress, the BH_2_/BH_4_ ratio was analyzed, as a marker of redox status. At Weeks 24 and 28, no differences in BH_2_/BH_4_ ratio could be observed between groups (Table 3). Levels of TAA were significantly increased in the LF group (*p* < 0.01) and appeared to be increased in the LF+ group (although not reaching significance) compared with HF and HF+ after 24 weeks. After 28 weeks, LF and LF+, compared with HF, showed significantly increased TAA levels (*p* < 0.05). 

### 3.4. Liver Status

Both LF and LF+ displayed significantly reduced liver weights (LW) for both time points (*p* < 0.01) compared with HF and HF+ (Table 3). No differences were detected in the hepatic antioxidants AA, SOD, GSH and GSSG, or in the oxidative stress markers L-arg, ADMA and BH_2_/BH_4_ ratio (Table 4 and Table 5). As a quantitative measure of steatosis, levels of hepatic TC and TG were analyzed. After 24 weeks, both LF and LF+ had significantly reduced hepatic TC levels compared with HF (*p* = 0.01). LF+ also showed reduced TC levels compared with HF+ (*p* = 0.0341). After 28 weeks, TC levels of both LF and LF+ were significantly reduced compared with HF and HF+ (*p* < 0.001). LF+ had reduced TG levels compared with HF (*p* = 0.0162) after 24 weeks, and showed decreased TG levels compared with HF and HF+ (*p* < 0.001) after 28 weeks. LF only showed reduced TG levels after 28 weeks compared with HF+ (*p* = 0.0006; Figure 2).

### 3.5. Histopathological Evaluation

**Figure 3:**
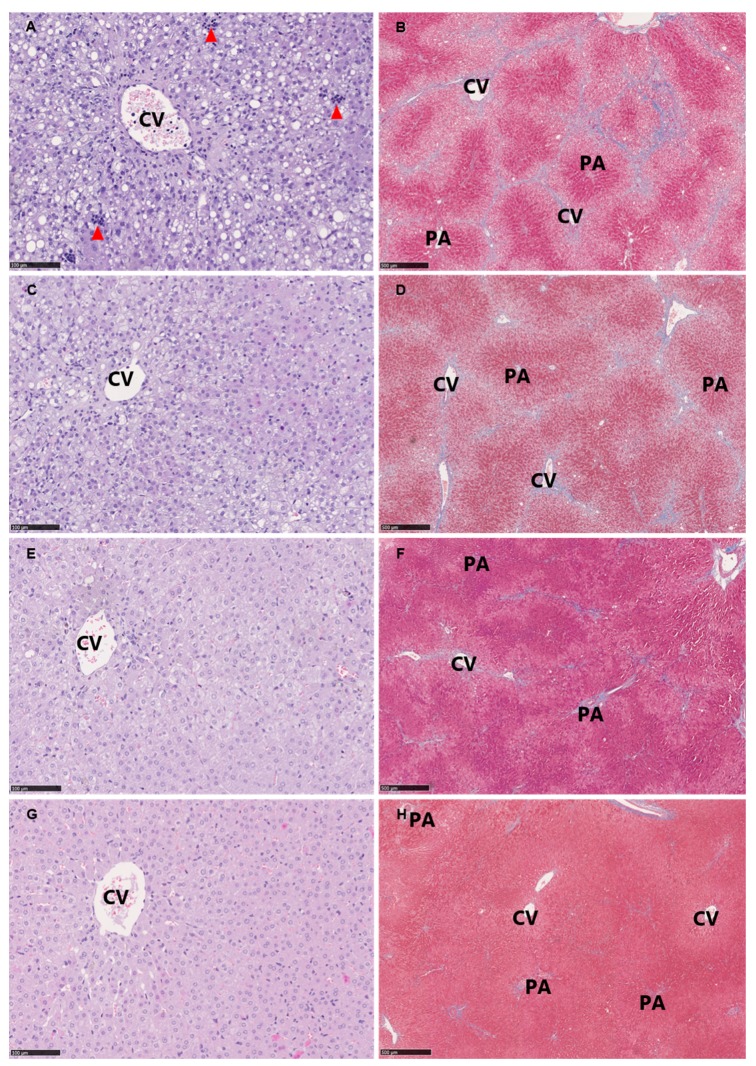
Representative histological images at study termination (Week 28) of hepatic tissue from: HF (**A**,**B**); HF+ (**C**,**D**); LF (**E**,**F**); and LF+ (**G**,**H**). (**A**,**C**,**E**,**G**) Hematoxylin and eosin stain, scale bar 100 µm. (**B,D,F,H**) Masson’s trichrome stain (fibrosis stained blue), scale bar 500 µm. Classical pathological lesions of NASH include: steatosis distributed as macro and micro vesicular steatosis, lobular inflammation as inflammatory clusters (red arrowheads) and ballooning hepatocytes (not clearly identified in the current magnification). Advanced and bridging fibrosis is seen in (**B,D**) (fibrosis in blue). CV, central vein; PA, portal area.

**Figure 4:**
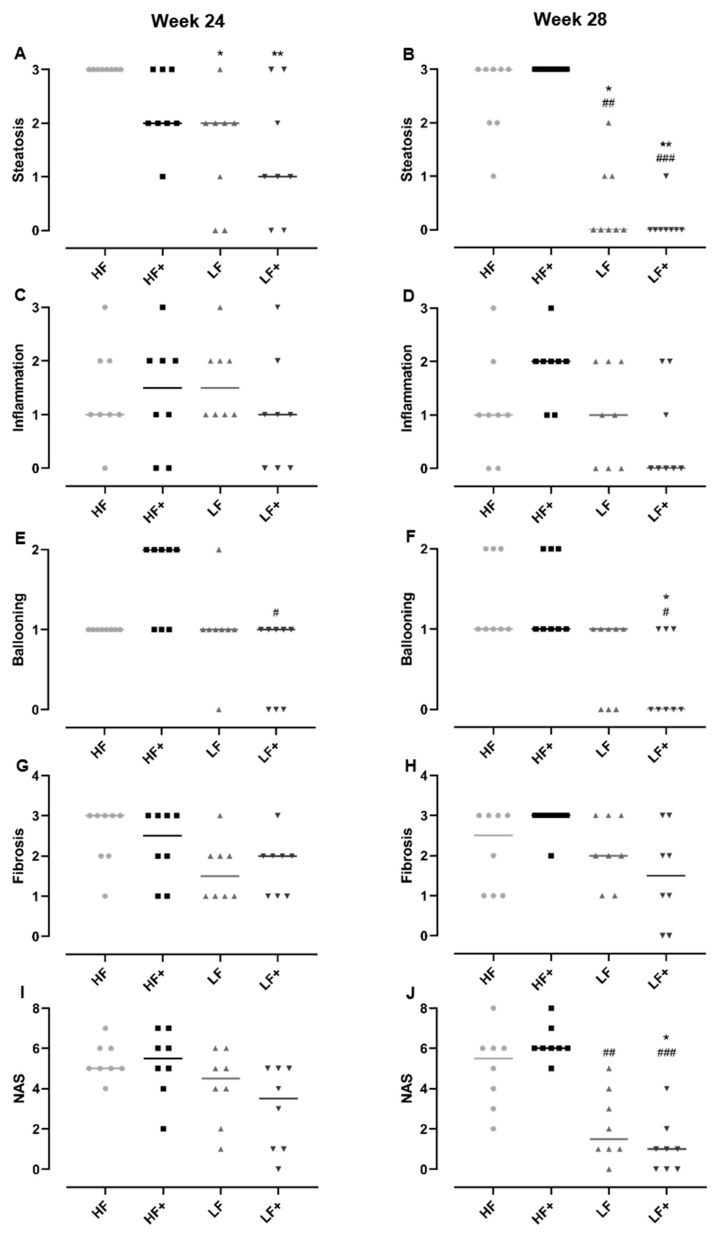
Histopathological scoring by the NAFLD activity scoring system and evaluation of disease severity. (**A**,**B**) Dietary intervention (LF) and dietary intervention combined with atorvastatin and vitamin E (LF+) reduced hepatic steatosis compared to HF (after 24 and 28 weeks) and HF+ (after 28 weeks), with increasing significance in the LF+ group. (**C**,**D**) No significant effect was observed after 24 and 28 weeks in the inflammatory score. However, a trend towards reduction in the LF+ group compared with HF and HF+ was seen. (**E**,**F**) A significant reduced ballooning score in the LF+ group compared with HF+ were detected after 24 and 28 weeks. Furthermore, after 28 weeks LF+ were also significantly reduced compared with the HF group. The LF group displayed no effect compared to HF and HF+. (**G**,**H**) After 28 weeks, the fibrosis score of the LF+ appeared to be decreasing compared with HF and HF+. However, no significant effects were detected. (**I**,**J**) No significant differences in the NAS were detected after 24 weeks. After 28 weeks, both LF and LF+ were significantly reduced compared with HF+, while LF+ were also significantly reduced compared with HF, with no guinea pig reaching the minimum score of 5. Data are presented as individual values with medians. * *p* < 0.05, ** *p* < 0.01, compared to HF; # *p* < 0.05, ## *p* < 0.01, ### *p* < 0.001, compared to HF+.

As expected, the HF diet induced histopathological lesions hallmarking NASH, e.g. steatosis, lobular inflammation and ballooning hepatocytes as well as fibrosis progressing to bridging fibrosis (Figure 3). Atorvastatin and vitE therapy and/or diet change decreased steatosis compared with HF (*p* < 0.05) after 24 weeks. After 28 weeks, LF and LF+ showed reduced steatosis compared with both HF and HF+ (*p* < 0.05) with increasing significance for the LF+ group (*p* < 0.0037 vs. *p* < 0.0245). Ballooning hepatocytes were only significantly affected when the diet change was combined with atorvastatin and vitE therapy, compared with HF+ (*p* = 0.0023) after 24 weeks, and compared with HF and HF+ (*p* = 0.0158) after 28 weeks. No significant effect on inflammation and fibrosis was detected, although a trend towards a reduction in the LF+ group could be seen (*p* = 0.063 and *p* = 0.0501 for inflammation and fibrosis, respectively; Figure 3 and Figure 4) after 28 weeks. The NAFLD activity score (NAS) is used as a tool in the assessment of disease severity of NASH, with a threshold value of ≥5 shown to correlate with the diagnosis of NASH [27]. The NAS is the combined score of steatosis, ballooning hepatocytes and lobular inflammation. After 28 weeks of treatment, the LF group had a lower NAS compared to HF+ (*p* = 0.0068), while the NAS of LF+ was significantly reduced compared with both HF and HF+ (*p* < 0.05) and not a single animal reaching the score of 5 (Figure 4). At Week 28, liver morphology was improved in LF and LF+ (median score of 2) compared to HF+ (median score of 4, *p* < 0.01), but not compared to HF (median score of 3) (data not shown).

### 3.6. Expression of Target Genes/qPCR

**Figure 5:**
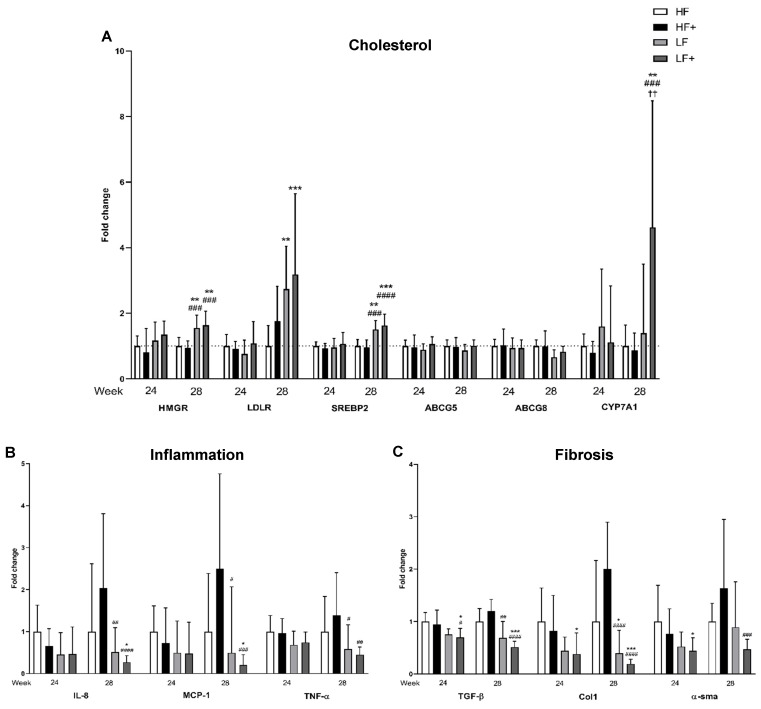
(**A**) Expression of HMGR and SREBP2 was increased in the LF and LF+ group compared with HF and HF+, while an increase in LDLR expression was detected in the LF and LF+ group compared to HF after 28 weeks. LF+ had increased CYP7A1 expression compared to HF, HF+ and LF at Week 28. No differences were detected in the expression of bile acid transporters, ABCG5 and ABCG8, at any time point. (**B**) Compared to HF, a decrease in expression of IL-8 and MCP-1 was only detected in the LF+ group. LF and LF+ were both different in IL-8 and MCP-1 expression compared to HF+, after 28 weeks. At Week 28, TNF-α expression was reduced in the LF and LF+ group compared to HF+, although a non-significant decrease, compared to HF, could also be observed. (**C**) After 24 weeks, LF+ showed reduced expression in TGF-β compared to HF and HF+, with increased reduction after 28 weeks and LF was reduced compared to HF+ after 28 weeks. LF and LF+ both showed reduction in Col1a1 expression after 28 weeks, with increased significance in the LF+ group. LF+ also showed reduction in Col1a1 after 24 weeks, compared to HF. α-sma expression in LF+ was reduced after 24 weeks compared to HF and after 28 weeks compared to HF+. Data are presented as means with ranges expressed as fold-changes relative to HF. * *p* < 0.05, ** *p* < 0.01, *** *p* < 0.001, compared to HF; # *p* < 0.05, ## *p* < 0.01, ### *p* < 0.001, #### *p* < 0.0001; compared to HF+. †† *p* < 0.01 compared to LF; *n* = 6–8 (*n* = 6 for HF for all genes due to practical measures and to avoid plate to plate variation in the qPCR assay). HMGR, 3-hydroxy-3-methylglutaryl-coenzyme A reductase; LDLR, low-density lipoprotein receptor; SREBP2, sterol regulatory element binding protein 2; ABCG5 and -8, ATP-binding cassette sub-family G member 5/8; CYP7A1, cytochrome P450 7A1; IL-8, interleukin-8; MCP-1, monocyte chemotactic protein 1; TNF-α, tumor necrosis factor α; TGF-β, transforming growth factor β; Col1a1, collagen 1; α-sma, α smooth muscle actin.

As a measure of the effect of pharmacological and/or dietary intervention, target genes of cholesterol uptake, synthesis and excretion were analyzed. After 24 weeks, no differences between groups were detected in the expression of HMGR, low-density lipoprotein receptor (LDLR), sterol regulatory element binding protein 2 (SREBP2), ATP-binding cassette sub-family G member (ABCG5 and ABCG8) or cytochrome P450 7A1 (CYP7A1). When the study continued for 28 weeks, SREBP2, a main regulator of cholesterol metabolism, was upregulated in LF and LF+ compared with HF and HF+ (*p* < 0.01) with 0.5–0.6-fold. SREBP2 induces LDLR, which increases the uptake of cholesterol from the circulation. An upregulated LDLR expression was observed in LF and LF+ that both showed a significantly 1.7–2.1-fold increased expression compared with HF (*p* < 0.01). HMGR is the rate limiting enzyme in the synthesis of cholesterol and was shown to be increased in LF and LF+ compared with HF and HF+ (*p* < 0.01). In the excretion of cholesterol, bile acid synthesis and transporters for export of bile acid are activated. CYP7A1 is expressed in relation to bile acid synthesis and was here shown to be significantly increased in the LF+ group compared with HF, HF+ and LF (*p* < 0.01) with 3.6-, 3.8- and 3.3-fold respectively. Expression of the transporter gene ABCG5 was similar between groups, while expression of ABCG8 was only significantly different between LF and HF+ (*p* = 0.0407; Figure 5). Inflammation is used in the diagnosis of NASH and is linked to the development of fibrosis. Here, we showed that after 28 weeks only the LF+ group was significantly reduced compared with the HF group in the expression of the inflammatory markers IL-8 and monocyte chemotactic protein 1 (MCP-1) (*p* < 0.05) with 1.25-fold, while both LF and LF+ were decreased compared with HF+ (*p* < 0.05). The expression of TNF-α was decreased in LF and LF+ compared with HF and HF+, although differences were only statistically significant when compared to HF+ (*p* < 0.05). No differences were detected after 24 weeks, although a trend towards a reduction in IL-8, MCP-1 and TNF-α could be seen in the LF and LF+ groups compared with HF and HF+ (Figure 5). The consequence of a chronic inflammatory state is an increase in expression of pro-fibrotic growth factors, such as transforming growth factor β (TGF-β) [29]. After 24 weeks, the expression of TGF-β was only reduced when combining a dietary intervention with atorvastatin and vitE (the LF+ group), compared with HF and HF+ (*p* < 0.05). Continuing for 28 weeks resulted in the LF group becoming significantly reduced compared with HF+ (*p* = 0.0011) and an increase in significance in the LF+ group, with a two-fold down regulation, compared with HF and HF+ (*p* = 0.001). TGF-β activates quiescent hepatic stellate cells (HSC), which leads to extracellular matrix deposition and promotion of fibrosis [19]. To assess activated HSC and fibrosis formation, expression of α smooth muscle actin (α-sma) and collagen 1a1 (Col1a1) were analyzed. After 24 weeks, α-sma and Col1a1 were significantly reduced in the LF+ group compared with HF (*p* < 0.05). α-sma expression showed no differences in the LF group compared to HF and HF+ after 28 weeks, while the LF+ group displayed a 1.2-fold reduction compared with HF+ (*p* = 0.0003). Continuing for 28 weeks resulted in a significant decrease in Col1a1 expression in both LF and LF+ compared with HF and HF+ (*p* < 0.05; Figure 5) of 0.6–0.8- and 1.6–1.8-fold, respectively.

## 4. Discussion

The present study examined the effect of NASH standard care (i.e., atorvastatin and vitE combined with or without diet change) on NASH resolution in guinea pigs in a 2 × 2 factorial design, allowing for a detailed evaluation of the in vivo hepatic changes resulting from pharmacological therapy and diet. Combining atorvastatin and vitE therapy with dietary intervention induced positive effects on dyslipidemia, and histopathological hallmarks as well as alterations in the expression of key target genes. The dietary intervention alone showed beneficial effects; however, the combination of therapy and diet change further improved the positive outcomes on hallmarks of NASH in the guinea pig model. 

First-line therapy in patients with NASH encompasses dietary changes and lifestyle modifications [10]. Caloric restrictions and exercise has been shown to improve the histopathological features of NASH, with increasing weight loss leading to increased hepatic improvements [30]. However, for many patients achieving and maintaining a suitable weight loss can be difficult, underlining the need for pharmacotherapy. Although no therapy has yet been approved, vitE is recommended for NASH patients and is associated with improvements in histopathological lesions [31]. Alongside vitE supplementation, atorvastatin can be administered to target dyslipidemia in NASH patients and clinical studies have shown beneficial effects of statin treatment on dyslipidemia and markers of hepatic health in patients [32,33,34]. However, comparisons between studies are often challenging due to differences in, e.g., inclusion criterions and intervention time, dose and statin-type. A recent review of 12 clinical trials summarizes mixed outcomes of statin treatment for NALFD resolution, although several show beneficial effects at least on some markers of hepatic health, and highlights that none of the studies reports hepatic damage or worsening of NAFLD hallmarks associated with treatment [35]. To date, no studies have confirmed the efficacy of combination therapy with atorvastatin and vitE together with dietary/lifestyle modifications; however, studies of isolated effects of the interventions have been positive [12,15,30,36,37,38]. Overall, findings from the present study supports clinical trials reporting a beneficial role of statins and vitE treatment in NASH.

In the Pioglitazone versus Vitamin E versus Placebo for the Treatment of Nondiabetic Patients with Nonalcoholic Steatohepatitis (PIVENS) trial, vitE (800 IU for 96 weeks) effectively decreased levels of ALT and AST, and induced significant improvements in NASH histopathology [33]. In addition to the administration of vitE, participants were advised regarding lifestyle modifications, including dietary restriction, weight loss and exercise [39]. This is in concordance with the present findings, where combination therapy with atorvastatin and vitE and dietary intervention significantly decreased liver injury enzymes, indicating an improvement in hepatocellular health. Furthermore, the LF+ group showed significant improvement in hepatic steatosis, hepatocellular ballooning and in the summarized NAS, compared with the HF and HF+ group. A trend towards reduction in lobular inflammation was also noted for the LF+ animals, although it did not reach significance. The expression of inflammatory target genes showed a significant reduction in IL-8, MCP-1 and a non-significant reduction in TNF-α, after 28 weeks, indicating a damping of the inflammatory response in the LF+ group. In the group only subjected to dietary intervention, a tendency towards reduction in ALT, hepatocellular ballooning, NAS, IL-8 and MCP-1 was also seen, although not significantly different from the HF group, which indicates that diet restriction alone is not sufficient to resolve the disease. Contrary to the PIVENS trial [33], treatment with atorvastatin and vitE alone did not improve ALT and AST. An improvement of AST and ALT was also reported for NAFLD patients receiving 20 mg atorvastatin for 12 weeks, with no additional effect by supplementing the statin with 400 IU vitE and 1000 mg vitC [32], whereas others have reported a reduction in ALT but not AST following six months of statin treatment (10–20 mg) in NAFLD patients [34]. The histological findings from the HF+ animals in the present study supports a state of hepatic stress, and show an increase NAS-score compared to LF and LF+ reaching significance at Week 28. At this time-point, the liver in HF+ animals is still subjected to inflammation, steatosis and decaying hepatic cells (ballooning) compared to the improvement in LF and LF+ animals. In agreement, gene expression analyses indicated a disrupted regulation of cholesterol metabolism and LDL-uptake in HF+ animals. The resulting stress and damage to hepatic cells would likely lead to the observed high AST and ALT levels in plasma. 

The presence of hepatic fibrosis constitutes a strong prognostic marker of mortality [40]. In the present study, the LF+ group showed a tendency to decreased fibrosis score supported by significant reduction in expression of fibrotic target genes (TGF-β and Col1a1). Varying results regarding fibrosis and statin or vitE therapy have been recorded [15,33,35,36], and both compounds have been associated with a decreased expression of TGF-β in animal studies and clinical trials [41,42]. As TGF-β is a strong activator of hepatic stellate cells and promoter of hepatic fibrosis [43], a decreased expression would be expected to reduce fibrosis. This correlates with our results of an increased effect on fibrotic markers when combining atorvastatin and vitE with a diet change.

Combination therapy with dietary intervention did not increase effects on plasma or hepatic cholesterol, the LF and LF+ group showed significantly reduced plasma levels of TC. As both atorvastatin and lifestyle modifications targets dyslipidemia, the lack of an increased effect by combination therapy could be expected [12,37,38]. The increased expression of HMGR, which is the target of atorvastatin, in LF and LF+ animals indicates that this may be mediated primarily by the change in diet as opposed to a more direct effect by atorvastatin and vitE. All groups displayed comparable bodyweights during the study. As guinea pigs regulate their feed intake by energy amount this is not surprising, and we have previously shown this to be the case also in the HF fed guinea pig model [3,18]. The induced dyslipidemia is clearly evident as are the hepatic consequences, constituting the primary end-points of the current study. 

Levels of plasma TG appeared to increase over time while hepatic TG levels were reduced in the LF+ group compared to HF controls, and non-significantly reduced in the LF group. It could be speculated that the increased plasma levels of TG is caused by the liver secreting excess amounts of TG in very low-density lipoproteins (VLDL) into the circulation. In the VLDL particles, TG is degraded converting the lipoprotein into LDL particles. The LDL particles are removed from the circulation by LDLR and determined for degradation or storage in the liver [44]. This is supported by the current finding of an increased expression of LDLR and SREBP2 (a major promoter of LDLR expression [45]) in the LF+ and LF animals. Although hydrolyzed TC is taken up by the liver, a decrease in hepatic TC levels was still observed. This could be explained by the increased expression in CYP7A1, promoting an increased conversion of cholesterol into bile acids and subsequent excretion [19], which is in agreement with the reduction in LW. Even though CYP7A1 gene expression in the LF group showed a small non-significant increase and the expression of ABCG5 and ABCG8 showed no increase, the effect on functional protein was not assessed. Thus, whether the subtle increase in mRNA transcription translated to alternations in functional protein cannot be concluded for the current study. 

It has been suggested that a HF diet increases the level of vitE in plasma due to the molecule’s lipid solubility and transportation in chylomicrons, LDL and HDL lipoproteins [46,47]. In the present study, elevated plasma αToc levels were observed in animals fed a HF diet compared with animals fed a LF diet, but this did not affect the hepatic αToc status. Thus, hepatic vitE accumulation was independent of dietary fat, suggesting that the plasma concentration of αToc is a poor surrogate marker of liver vitE status, a finding that may prove to have clinical relevance. Indeed, a very recent clinical study of the absorption kinetics of αToc in a small group of volunteers, found that the bioavailability of vitE was independent of dietary fat [48].

Throughout the present study, no significant positive effect was recorded for the HF+ group. A beneficial effect of the atorvastatin and vitE treatment was only obtained when therapy was combined with dietary intervention, implying that lifestyle modifications are a necessary part of treatment efficiency. However, as several non-significant trends towards improvement were observed in the dataset, it is possible that the intervention period was not sufficiently long for a significant resolution to manifest itself. 

In conclusion, dyslipidemic and hepatic biochemical markers were significantly reduced as a result of dietary intervention. Expression of fibrotic target genes significantly decreased following diet change combined with atorvastatin and vitE. Furthermore, inflammatory markers showed a tendency to reduction upon diet intervention, but only reached significance when combined with atorvastatin and vitE. The histopathological scoring and examination of hepatic tissue showed significant reduction in hepatic lesions and NAS with atorvastatin and vitE therapy combined with dietary intervention. These effects were evident after an intervention period of eight weeks, which correlates with the increased hepatic αToc levels at the given time. Our results suggest that dietary intervention induces positive effects on dyslipidemia, hepatic steatosis, inflammation, ballooning and fibrosis in NASH and that these are further improved by concurrent atorvastatin and vitE supplementation.

## Figures and Tables

**Table 1:** Dietary composition.

Nutrient	HF	HF+	LF	LF+
Protein (%)	16.7	16.7	16.8	16.8
Carbohydrates (%)	37.9	37.9	47.1	47.1
Fat (%)	20	20	4	4
Cholesterol (%)	0.35	0.35	-	-
Sucrose (%)	15	15	-	-
Vitamin E (all-rac-alpha-tocopheryl acetate (mg/kg feed))	125	375	125	375
Atorvastatin (mg/kg feed)	-	20	-	20

HF, high-fat diet; HF+, high-fat diet supplemented with atorvastatin and vitamin E; LF, chow diet; LF+, chow diet supplemented with atorvastatin and vitamin E.

**Table 2:** qPCR primer details.

Gene	Accession no.	Forward (5’-3’)	Reverse (3’-5’)	Product (bp)
*HMGR*	XM_003461336.3	TACAGACATGGGCATTGG GT	GGCAGGGAAAGTGTTGAGTG	184
*LDLR*	XM_013149927.1	GACGTGTCCCAGAGG AAGAT	CGAGTCGGTCCAGTAGATGTT	144
*SREBP2*	XM_003470391.3	GGGGCTCAAAGGTCTTCTCT	AGGACCCCATCAAAGTGAGG	189
*ABCG5*	XM_003472925.3	ATCCTGAGGCTGCTCGATTT	CCAGATCCAATCAGCAACCC	163
*ABCG8*	XM_003472926.2	GTCTCAACTCCCACCCTCTC	CTGAAGGGTCTTCTCCGAGG	170
*CYP7a1*	GQ507494.1	CTGGAGAAGGCAGGTCAACA	CTCCTTAGCTGTCCGGATGT	150
*IL-8*	NM_001173399.2	GGCAGCCTTCCTGCTCTCT	CAGCTCCGAGACCAACTTTGT	67
*MCP-1*	NM_001172926.1	TGCCAAACTGGACCAGAGAA	CGAATGTTCAAAGGCTTTGAAGT	75
*TNF-α*	NM_001173025.1	GCCGTCTCCTACCCGGAAAA	TAGATCTGCCCGGAATCGGC	203
*TGF-β*	NM_001173023.1	AACCCGAGCCGGACTACTATG	TGCTTTTATAGATATTGTGGCTGT TGT	78
*Col1a1*	XM_003466865.2	CTGGACAGCGTGGTGTAGTC	TCCAGAAGGACCTTGTTTGC	104
*α-sma*	ENSCPOT00000011693.2	GACATCAAGGAGAAGCTGTG	GCTGTTGTAGGTGGTTTCAT	273

HMGR, 3-hydroxy-3-methylglutaryl-coenzyme A reductase; LDLR, low-density lipoprotein receptor; SREBP2, sterol regulatory element binding protein 2; ABCG5/8, ATP-binding cassette sub-family G member 5/8; CYP7a1, cytochrome P450 7A1; IL-8, interleukin 8; MCP-1, monocyte chemotactic protein 1; TNF-α, tumor necrosis factor α; TGF-β, transforming growth factor β; Col1a1, collagen 1a1; α-sma, α smooth muscle actin.

**Table 3:** Plasma values at Pre-intervention, Week 24 and Week 28.

	Pre-intervention	Week 24				Week 28			
		HF	HF+	LF	LF+	HF	HF+	LF	LF+
FFA (mmol/L)	0.41 ± 0.04	0.64 ± 0.17	0.40 ± 0.10	0.63 ± 0.20	0.61 ±0.16	0.50 ± 0.21	0.42 ± 0.14	0.58 ± 0.20	0.42 ± 0.17
ALP (U/L)	46.13 ± 9.73	37.38 ± 7.76	40.0 ± 7.60	48.0 ± 9.32	47.25 ± 12.61	41.90 ± 8.36	38.5 ± 7.35	51.88 ± 10.66	50.13 ± 13.81
LW (%) ^j^	4.15 (3.44–4.86)	5.15 (4.58−5.72)	4.82 (4.10−5.54)	3.34 (2.91−3.77) ^f, h^	3.18 (2.73−3.63) ^f, i^	4.60 (3.64−5.56)	5.96 (4.76−7.16)	3.15 (2.81−3.50) ^e, i^	2.85 (2.53−3.16) ^b, f, i^
BH_2_/BH_4_	0.05 ± 0.02	0.13 ± 0.037 ^c^	0.11 ± 0.03 ^a^	0.13 ± 0.03 ^c^	0.12 ± 0.04 ^b^	0.08 ± 0.04	0.10 ± 0.04	0.08 ± 0.03	0.07 ± 0.03
TAA (µM)	40.56 ± 17.09	37.39 ± 14.40	32.28 ± 12.93	70.73 ± 27.94 ^a, e, i^	57.64 ± 19.34	28.69 ± 6.92	33.85 ± 15.51	55.05 ± 11.11 ^d^	56.51 ± 10.56 ^d^
αToc (µM) ^j^	4.52 (2.84−6.20)	3.10 (1.91−4.29)	6.70 (3.37−10.03) ^d^	1.35 (0.89−1.82) ^b, i^	1.89 (1.32−2.46) ^i^	2.70 (1.73−3.68)	6.63 (2.88−10.38) ^d^	1.59 (0.94−2.23) ^a, h^	2.69 (1.62−3.77) ^g^

FFA, free fatty acid; ALP, alkaline phosphatase; LW, liver weight; BH_2_/BH_4_, dihydrobiopterin/tetrahydrobiopterin ratio; AA, ascorbic acid; DHA, dehydroascorbate; α-Toc, alpha-tocopherol. Data are presented as mean ± standard deviation.^j^ Data are presented as geometric mean with 95 confidence intervals. ^a^ *p <* 0.05, ^b^ *p <* 0.01, ^c^ *p <* 0.001, compared with preintervention.^d^ *p <* 0.05, ^e^ *p <* 0.01, ^f^ *p <* 0.001, compared with HF.^g^ *p <* 0.05 ^h^ *p <* 0.01, ^i^ *p <* 0.001, compared with HF+.

**Table 4:** Liver status, oxidative stress markers. Pre-intervention and Week 24.

	Pre-intervention	HF	HF+	LF	LF+
AA (nmol/g)	1283.5 ± 379.51	1343 ± 533.44	1230.88 ± 474.33	1381.5 ± 518.21	1663.13 ± 598.48
BH_2_/BH_4_	0.21 ± 0.05	0.15 ± 0.10	0.23 ± 0.11	0.20 ± 0.12	0.23 ± 0.12
L-arg (nmol/g)	32.96 ± 8.73	30.47 ± 11.83	27.54 ± 7.10	29.14 ± 13.40	21.60 ± 6.54
ADMA (nmol/g)	0.73 ± 0.92	0.40 ± 0.53	0.92 ± 1.20	0.50 ± 0.53	0.17 ± 0.50
GSH (nmol/g)	3160.63 ± 503.63	3131.5 ± 316.22	2967.75 ± 336.71	3305.90 ± 498.19	3118.00 ± 424.33
GSSG (nmol/g)	317.72 ± 49.40	331.40 ± 51.99	293.81 ± 83.70	315.01 ± 61.90	307.70 ± 49.51
SOD (U/mg)	2489.5 ± 432.10	2621.40 ± 343.50	2495.00 ± 782.92	2449.40 ± 405.90	3107.80 ± 952.75
αToc (nmol/g) ^j^	1.71 (1.22–2.19)	3.47 (0.92–6.03)	5.14 (2.02–8.26) ^a^	3.39 (1.88–4.91)	3.30 (1.53–5.06)

AA, ascorbic acid; BH_2_/BH_4_, dihydrobiopterin/tetrahydrobiopterin ratio; L-arg, L-arginine; ADMA, asymmetric dimethylarginine; GSH, glutathione; GSSG, oxidized glutathione; MDA, malondialdehyde; SOD, superoxide dismutase; α-Toc, alpha-tocopherol. Data are presented as mean ± standard deviation. ^j^ Data are presented as geometric mean with 95 confidence intervals. ^a^ *p <* 0.05, compared with Pre-intervention.

**Table 5:** Liver status, oxidative stress markers. Pre-intervention and Week 28.

	Pre-intervention	HF	HF+	LF	LF+
AA (nmol/g)	1283.5 ± 379.51	1404.5 ± 497.99	1357 ± 457.91	1287.75 ± 573.73	1770 ± 380.30
BH_2_/BH_4_	0.21 ± 0.05	0.20 ± 0.12	0.30 ± 0.16	0.23 ± 0.10	0.23 ± 0.20
L-arg (nmol/g)	32.96 ± 8.73	22.10 ± 9.56	24.95 ± 6.47	23.60 ± 6.50	36.74 ± 10.80
ADMA (nmol/g)	0.73 ± 0.92	0.99 ± 0.80	0.70 ± 0.60	0.50 ± 0.60	0.91 ± 1.10
GSH (nmol/g)	3160.63 ± 503.63	3440.40 ± 487.40	3138.63 ± 301.32	3072.25 ± 305.80	3303.25 ± 170.97
GSSG (nmol/g)	317.72 ± 49.40	300.52 ± 69.90	295.40 ± 68.50	322.70 ± 32.30	324.80 ± 61.96
SOD (U/mg)	2489.5 ± 432.10	2783.90 ± 786.60	2512.00 ± 642.80	3206.40 ± 1059.80	2899.13 ± 502.82
αToc (nmol/g) ^j^	1.71 (1.22–2.19)	2.87 (0.90–4.84)	9.43 (3.68–15.18) ^a, b, c^	3.49 (1.95–5.02)	8.61 (4.98–12.24) ^a, b, c^

AA, ascorbic acid; BH_2_/BH_4_, dihydrobiopterin/tetrahydrobiopterin ratio; L-arg, L-arginine; ADMA, asymmetric dimethylarginine; GSH, glutathione; GSSG, oxidized glutathione; MDA, malondialdehyde; SOD, superoxide dismutase; α-Toc, alpha-tocopherol. Data are presented as mean ± standard deviation. ^j^ Data are presented as geometric mean with 95 confidence intervals. ^a^ *p <* 0.001, compared with Pre-intervention. ^b^ *p <* 0.01, compared with HF. ^c^ *p <* 0.05, compared with LF.
